# Synaptic vesicle pool heterogeneity drives an anomalous form of synaptic plasticity

**DOI:** 10.1073/pnas.2401734121

**Published:** 2024-02-29

**Authors:** Natalie J. Guzikowski, Ege T. Kavalali

**Affiliations:** ^a^Department of Pharmacology, Vanderbilt University, Nashville, TN 37240-7933; ^b^Vanderbilt Brain Institute, Vanderbilt University, Nashville, TN 37240-7933

Chemical neurotransmission begins when synaptic vesicles fuse with the plasma membrane at the presynaptic active zone and release their neurotransmitters. However, before synaptic vesicles can fuse and release their content they must go through multiple regulatory steps; they must translocate to the active zone, dock at a fusion site, and then become biochemically primed to ultimately undergo fusion (1). Classical models assume that all synaptic vesicles within a synapse comprise a homogenous population that can equally complete these steps and have the same potential to fuse, even under different functional contexts. In this model, functional differences among synaptic vesicle populations are ascribed to their spatial distribution within the synapse relative to the active zone or to voltage-gated Ca^2+^ channels that deliver action potential mediated Ca^2+^ influx. This premise is supported by the routine observation that all synaptic vesicles look similar in electron micrographs, with a small fraction docked at the active zone, while others are at a distance.

However, an increasing number of findings within the last two decades have been difficult to reconcile with this simple picture. For instance, studies have demonstrated a significant functional and molecular dichotomy between synaptic vesicles that fuse spontaneously versus those that fuse in response to presynaptic action potentials. Indicating that some vesicles have a higher propensity for spontaneous fusion but are not as readily available for evoked release and others prefer to fuse in response to rapid Ca^2+^ transients and are more reluctant to fuse spontaneously (2). Furthermore, an increasing number of studies have uncovered that single synaptic terminals release multiple neurotransmitters often from non-overlapping pools of synaptic vesicles (3). Finally, molecular dissection of kinetically distinct forms of evoked neurotransmission also suggests clear molecular differences between the pools of vesicles that carry out fast synchronous neurotransmission versus asynchronous neurotransmitter release (4). When taken together, these accumulating findings suggest an updated framework where synaptic vesicles are a heterogenous population of organelles, that possess intrinsic molecular differences and/or differential interaction partners that diversify their fusion propensity or neurotransmitter content. From a cell biological perspective, this diversity is not surprising as distinct vesicle trafficking pathways are known to generate vesicles with diverse functional properties within cells (5, 6).

In PNAS, Koppensteiner et al. (7) present an exquisitely detailed study demonstrating that a rather anomalous form of synaptic plasticity is driven by the recruitment of an additional molecularly distinct pool of vesicles. The authors analyze synaptic potentiation at medial habenula (MHb) terminals in the interpeduncular nucleus (IPN) where typically inhibitory GABA_B_ receptor activation elicits not only the potentiation of release but also the complete transformation of the synaptic release profile from tonic to phasic release. During tonic release, these synapses show facilitation that is sustained under moderate 10 Hz frequency stimulation, whereas during phasic release under the same conditions, an initial robust and larger response rapidly depresses to a smaller amount within seconds. This observation is within itself quite unusual as the dominance of either tonic or phasic release in a particular synapse is generally considered to be a hard-wired property, depending on synapse type and morphology, as well as the identity of presynaptic and postsynaptic neurons. However, in these MHb to IPN synapses, neuromodulatory inputs triggering GABA_B_ receptor activation appear to be sufficient to elicit a dramatic switch from a tonic to phasic release profile. In addition, this switch happens despite the fact that presynaptic GABA_B_ receptor activation is often inhibitory acting either via suppression of presynaptic voltage-gated Ca^2+^ channel gating (8) or via direct interactions of the G-proteins with the release machinery downstream of Ca^2+^ entry (9). However, MHb terminals show robust synaptic potentiation and phasic release upon GABA_B_ receptor activation due to a non-canonical signaling pathway that the authors could not ascribe to classical cAMP or DAG-PKC signaling mechanisms.

In PNAS, Koppensteiner et al. present an exquisitely detailed study demonstrating that a rather anomalous form of synaptic plasticity is driven by the recruitment of an additional molecularly distinct pool of vesicles.

Nevertheless, the authors embark on a thorough investigation of the synaptic mechanisms that may underlie such a dramatic phenotypic switch from facilitating tonic release to depressing phasic release. First, they demonstrate that the transition from tonic to phasic release profile requires augmentation of pre-synaptic Ca^2+^ influx and subsequent recruitment of synaptic vesicles to the readily releasable pool (RRP), as measured functionally and assessed ultrastructurally. Moreover, the authors find that while tonic release is maintained by synaptic vesicles that are tightly coupled to presynaptic Ca_v_2.3 voltage-gated Ca^2+^ channels (also called the R-type), vesicles recruited after GABA_B_ activation are only loosely coupled to these Ca^2+^ channels.

Next, to delineate the molecular players implicated in new vesicle recruitment to the RRP, the authors investigate the role of synaptoporin (SPO) and Ca^2+^-dependent activator protein for secretion2 (CAPS2), two understudied synaptic proteins that are relatively enriched in the MHb synaptic terminals. Using SPO knock-out mice they find that SPO is required for the modulation of the tonic release phenotype, although its loss does not alter the phenotypic switch. In contrast, CAPS2-deficient terminals from CAPS2 knock-out mice show that CAPS2 is critically required for the transformation of the tonic release profile to phasic release.

To gain further insight into these findings, the authors employ a novel ultrastructural visualization method; “flash-freeze fracture.” With flash-freeze fracture, the authors quantify the location of both SPO and CAPS2 relative to the active zone and synaptic vesicles directly following stimulation. These experiments reveal that while both SPO and CAPS2 co-exist in the same terminals, only CAPS2 translocates to the presynaptic active zone during phasic release and retains the RRP increase for several minutes. This flash-freeze fracture approach is a remarkable feat that provides a key technical advancement with both high spatial and temporal resolution, necessary to visualize the dynamic transformation that these synapses go through.

Interestingly, under the same conditions, Koppensteiner et al., observe the opposite regulation of spontaneous release in contrast to evoked neurotransmission. As detailed above, upon GABA_B_ receptor activation, evoked release is augmented coupled with a transition from tonic to phasic transmission, however, spontaneous release frequency is simultaneously decreased as typically seen with GABA_B_ regulation in other systems (9). This finding suggests that separable signaling mechanisms are mediated by the same receptor, that in turn differentially regulate evoked and spontaneous release within the same MHb-IPN synaptic terminals.

This study by Koppensteiner et al., begins to dissect the molecular mechanisms underlying this unique plasticity profile, however, some questions remain. For instance, the molecular mechanisms that underlie the roles of SPO and CAPS2 in synaptic vesicle pool dynamics are unknown. Importantly, the identity of CAPS2’s interaction partner on vesicles also remains to be determined. In addition, as indicated above, the exact signaling pathway that drives the potentiating effects downstream of GABA_B_ receptors is elusive and it is unclear how the same receptor pathway elicits opposite outcomes in the regulation of evoked versus spontaneous release at the same synapse. Despite these open questions, the notion that plasticity involves the recruitment of a new set of molecularly distinct vesicles to maintain the phenotypic switch associated with potentiation is unequivocal.

As discussed above, extremely divergent responses to activity such as sustained tonic release versus more transient phasic release are thought to be hard-wired properties of synapses, although synapses often lack clear ultrastructural correlates that predict a certain form of plasticity (10). Deviating from this notion, earlier studies in invertebrates as well as mammalian synapses have uncovered cases of robust transformations in plasticity dynamics and synaptic vesicle recycling pathways dependent on ambient activity and developmental stage (11–13). This is consistent with the premise that such differences are either associated with vesicle dynamics or intrinsic differences among synaptic vesicle pools. In these instances, adaptations at an individual synapse display a large functional range contradicting the premise that plasticity profiles and associated vesicle trafficking mechanisms are a hard-wired property.

**Fig. 1.**
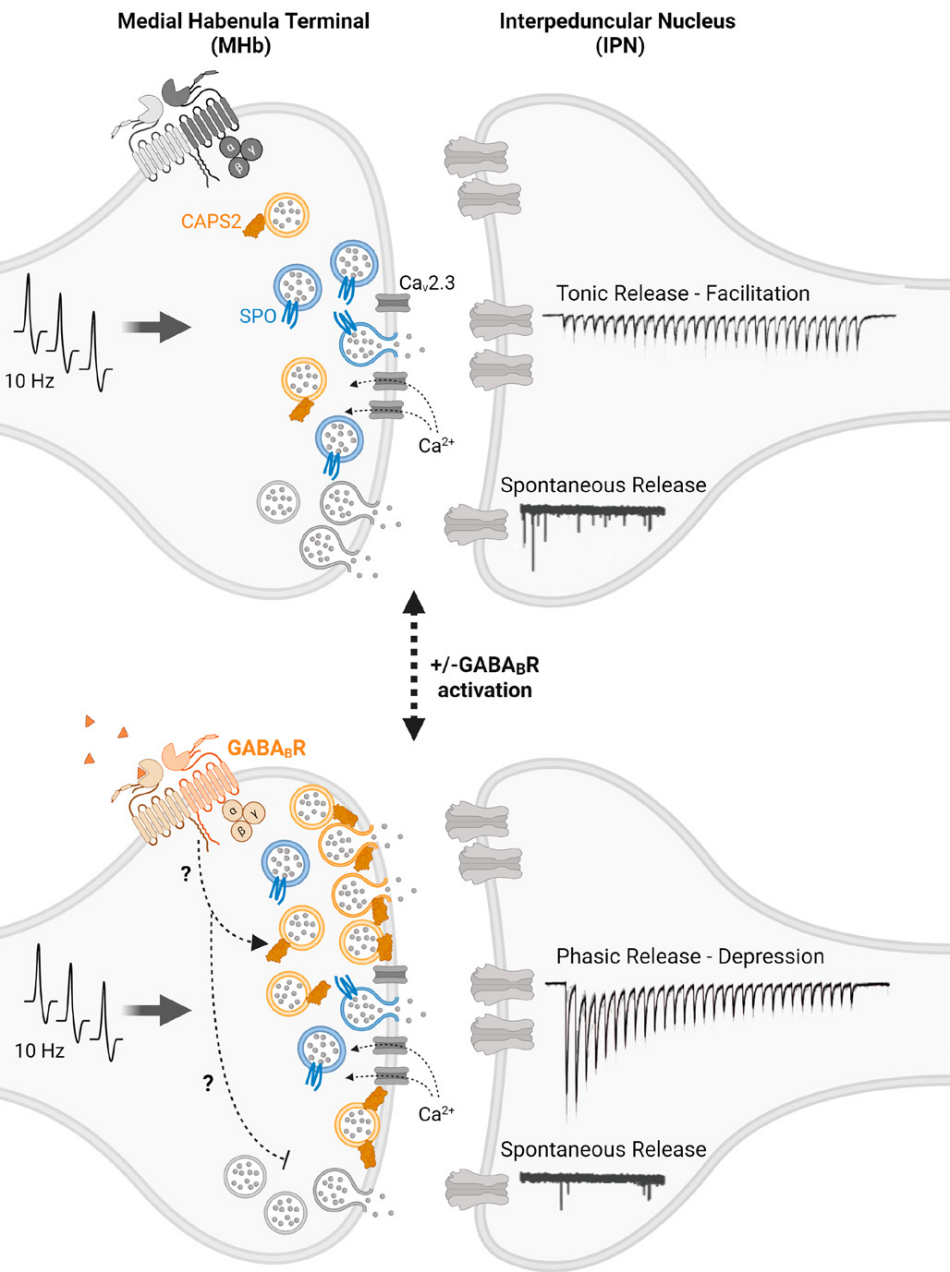
Model proposed by Koppensteiner et al. (7); moderate 10 Hz frequency stimulation leads to facilitating tonic release at MHb-IPN synapses, modulated by synaptoporin (SPO) associated synaptic vesicles. Upon GABA_B_ receptor activation, there is the subsequent recruitment of Ca^2+^-dependent activator protein for secretion2 (CAPS2) to the active zone with associated vesicles, augmenting the readily releasable pool (RRP), and altering the release profile to depressing phasic release. With GABA_B_ receptor activation, there is the opposite regulation of spontaneous release in contrast to evoked neurotransmission, with a simultaneous decrease in spontaneous release frequency.

Taken together, these findings indicate a complex molecular nano-organization within single synapses where synaptic vesicle molecular composition and localization are tightly regulated in a manner that impacts distinct forms of release and their plasticity (14). For instance, the investigation of the diversity of vesicle-associated SNARE proteins has provided great insight into how vesicular proteins can serve as molecular markers of synaptic vesicle sub-pools with different functional properties (6). Koppensteiner et al., expand this premise to additional molecular markers and demonstrate the utility of synaptic vesicle pool heterogeneity as a substrate for plasticity (Fig. 1).

Although as scientists we tend to be conservative and usually prefer classical simple models to account for our observations, the accumulation of well-documented anomalous facts—as provided here by Koppensteiner et al., —necessitates the development of more comprehensive paradigms that can account for these anomalies as well as canonical observations (15). Therefore, our broadening perspective on the structure and function of synapses that incorporates synaptic vesicle pool molecular diversity and nano-organization of the active zone will be increasingly needed as the norm rather than the exception to elucidate the full range of neurotransmitter signaling in the nervous system.
